# Is endotracheal intubation a non-beneficial treatment in patients with respiratory failure due to paraquat poisoning?

**DOI:** 10.1371/journal.pone.0195071

**Published:** 2018-03-28

**Authors:** Meng-Ruey Wu, Chia-Ying Hsiao, Chun-Han Cheng, Feng-Ching Liao, Chuan-Lei Chao, Chun-Yen Chen, Hung-I Yeh, Min-I Su

**Affiliations:** 1 Division of Nephrology, Department of Internal Medicine, MacKay Memorial Hospital, Taitung branch, Taiwan; 2 Division of Gastroenterology and Hepatology, Department of Internal Medicine, MacKay Memorial Hospital, Taitung branch, Taiwan; 3 Division of Cardiology, Department of Internal Medicine, MacKay Memorial Hospital, Taitung branch, Taiwan; 4 Division of Cardiology, Department of Internal Medicine, MacKay Memorial Hospital, Taipei, Taiwan; 5 MacKay Medical College, New Taipei City, Taiwan; National Yang-Ming University, TAIWAN

## Abstract

**Introduction:**

Paraquat poisoning can result in dysfunction of multiple organs, and pulmonary fibrosis with respiratory failure is the major cause of mortality. For terminally ill patients, some life-prolonging treatments can be non-beneficial treatments (NBT). The objective of this study was to determine if intubation is a NBT for patients with respiratory failure due to paraquat poisoning.

**Methods:**

The study included 68 patients with respiratory failure due to paraquat poisoning. Patients were hospitalized at MacKay Memorial Hospital, Taitung Branch, Taiwan, between 2005 to April 2016. Composite outcomes of intra-hospital mortality, the rate of do-not-resuscitate (DNR) orders, prescribed medications, length of stay, and medical costs were recorded and compared between the do-not-intubate (DNI) group and endotracheal intubation (EI) group.

**Results:**

Intra-hospital mortality rate for the entire population was 100%. There were significantly more patients with DNR orders in the DNI group (P = 0.007). There were no differences in the length of hospital stay. However, patients in DNI group had significantly less vasopressor use and more morphine use, shorter time in the intensive care unit, and fewer medical costs.

**Conclusion:**

The procedure of intubation in patients with respiratory failure due to paraquat poisoning can be considered inappropriate life-prolonging treatment.

## Introduction

Although the use of paraquat has been illegalized in many countries, it is still one of the major herbicides used in Taiwan. It is frequently ingested owing to its unrestricted availability. Paraquat ingestion occurs frequently in the agricultural countryside, either accidentally or as a suicide attempt [1]. The high toxicity of paraquat results in extremely high mortality [2]. Between January 2001 and December 2002, a prospective study in two medical centers in southern Taiwan, reported 63 poison-related fatalities; most were determined to be suicide (92.1%; 58/63). Paraquat was the major agent involved in fatalities (46.3%; 31/63) [3]. After the sale of paraquat was banned in South Koreain end-October 2012, the pesticide suicide mortality rate halved, from 5.26 to 2.67 per 100 000 population, between 2011 and 2013 [4]. The overall pesticide suicide mortality rate in 2013 was also significantly lower than expected, based on previous trends from 2003 to 2011 (Rate ratio = 0.63, 95% CI 0.55 to 0.73).

Owing to the fulminant inflammatory reactions, paraquat poisoning causes systemic toxicity and multiple organ dysfunction [5]. The primary cause of mortality is pulmonary fibrosis and respiratory failure [6]. The therapeutic regimen with immunosuppressive agents applied in patients with severe lung injury secondary to systemic lupus erythematosus [7, 8] was therefore referred in patients with paraquat poisoning. Lin et al. [9, 10] demonstrated that simultaneous pulse therapy using cyclophosphamide (CP) and methylprednisolone (MP), followed by dexamethasone, resulted in lower mortality when compared to the conventionally treated control group. Activated charcoal hemoperfusion was also used to decrease the concentration of paraquat in plasma [11, 12]. In 2014, Wu et al. [13] analyzed data from 1997 to 2009, retrieved from the National Health Insurance Research Database (NHIRD) of Taiwan. In this study, of the 1811 patients hospitalized for paraquat poisoning, mortality was 78.6%, and 1018 patients (56.2%) developed respiratory failure among which, mortality was 93.3% (950/1018).

Since paraquat poisoning has a high mortality after progression to respiratory failure, the possibility of treatment futility, or non-beneficial treatment (NBT) should be considered for these patients. NBT is defined as an inappropriate life-prolonging treatment and can be seen as a disservice to patients who are subjected to ongoing, and likely uncomfortable, conditions without direct benefit [14]. Owing to the government policy, there is increased awareness that advanced life-prolonging treatments for terminally ill patients may not be beneficial. However, the term “terminally ill patients” is usually limited to patients with cancer, stroke, chronic heart failure, and chronic kidney, liver, and obstructive pulmonary diseases. It is not clear if patients with acute paraquat poisoning are suitable candidates for advanced life-prolonging treatments, like intubation while respiratory failure develops. Endotracheal intubation for patients with respiratory failure is the most common and intuitive resuscitative procedure. However, in patients with paraquat poisoning, we cannot make sure if it is one kind of NBT, which means this advanced life-prolonging treatment would result in a quality of life that the patients have previously stated they would not wish [15].

Given physicians’ concerns about providing NBT to patients with paraquat poisoning, this study explores the outcomes of patients who progressed to respiratory failure. The outcomes of patients who chose to sign a do-not-intubate (DNI) order and of those who chose to have endotracheal intubation (EI) were compared, with a particular focus on whether intubation is an NBT or not.

## Materials and methods

### Subjects

This retrospective observational study was conducted in accordance with the Declaration of Helsinki and approved by the Institutional Review Board of MacKay Memorial Hospital. The patient records and information were anonymized and de-identified prior to analysis. Between 2005 to April 2016, 90 consecutive patients with paraquat ingestion were admitted to the Taitung Branch at MacKay Memorial Hospital, Taiwan. Among them, 68 patients who progressed to respiratory failure were identified for this study, and segregated into two groups: the do-not-intubate (DNI) group and endotracheal intubation (EI) group. The definition of respiratory failure means a condition of respiratory insufficiency (eq. inadequate oxygen saturation or paradoxical breathing) requiring intubation and mechanical ventilation, regardless of the fraction of inspired oxygen. Medical history, clinical signs, and laboratory examinations were used to diagnose paraquat poisoning. Without a spectrophotometer to measure plasma paraquat concentration, a qualitative urine-sodium dithionite reaction was used. Demographic data, do-not-resuscitate (DNR) and DNI orders, prescribed medications, length of stay, and medical costs were obtained from the hospital medical registry.

### Clinical outcomes

The outcome measure in the current analysis was the time from paraquat poisoning until the first component of the composite endpoint: intra-hospital mortality, the rate of DNR orders, prescribed medications, length of stay in the intensive care unit (ICU) and in the hospital, and medical costs.

### Statistical analysis

Results are expressed as the mean ± standard deviation or as percentages. Student’s *t-*test was used to compare differences between groups for continuous variables, and the chi-square test was employed for categorical data. A p-value <0.05 was considered significant. All statistical analyses were performed using the SPSS software, version 22 (IBM SPSS Statistics, Armonk, NY).

## Results

### Patient characteristics

Baseline clinical characteristics of patients with respiratory failure are shown in Table 1. The mean age of the 68 patients developing respiratory failure after paraquat intoxication was 57.72 ± 17.42 years, with 47 (69.1%) male and 21 female. All patients (100%, 68/68) died in the hospital. On the other hand, the exluded 22 patients who did not suffer respiratory failure had all survived and discharged from hospital. Sixty-two patients (91.2%) signed DNR consent, consequently, only 6 patients (8.8%) underwent cardiopulmonary resuscitation before the death. The proportion of patients receiving pulse therapy of immunosuppressive agents, MP and CP, and hemoperfusion was 89.5%. The mean length of stay in the ICU, and in the hospital, were 62.41 ± 86.12 hours, and 74.69 ± 120.07 hours, respectively.

**Table 1:** Baseline characteristics of the patients with respiratory failure by paraquat poisoning (n = 68).

Variable	All Patients
Male (%)	47 (69.1)
Age (years)	57.72 ± 17.42
Creatinine (mg/dL)	2.02 ± 2.23
AST (IU/L)	63.46 ± 67.47
DNR orders (%)	62 (91.2)
CPR (%)	6 (8.8)
DNI (%)	36 (52.9)
EI (%)	32 (47.1)
MP + CP (%)	61 (89.7)
HP (%)	61 (89.7)
Intra-hospital mortality (%)	68 (100)
LOS in ICU (hours)	62.41 ± 86.12
LOS in hospital (hours)	74.69 ± 120.07

Abbreviations: AST, Asparte aminotransferase; DNR, do-not-resuscitate; CPR, cardiopulmonary resuscitation; DNI, do-not-intubation; EI, endotracheal intubation; MP + CP, pulse therapy of methylprednisolone and cyclophosphamide; HP, hemoperfusion; LOS, length of stay; ICU, intensive care unit

### Differences between DNI and EI groups

Of the 68 patients progressing to respiratory failure, 36 (52.9%) chose not to be intubated and 32 patients (47.1%) chose to be intubated (Table 2). There were no significant differences in the percentage of the patients who received pulse therapy of immunosuppressive agents and hemoperfusion between DNI and EI groups (91.7%, 33/36 vs. 87.5%, 28/32, P = 0.57). The percentage of DNR orders signed before death was significantly greater in the DNI group than in the EI group (100%, 36/36 vs. 81.3%, 26/32, P = 0.007). Although the use of vasopressors in patients with an unstable hemodynamic profile is acceptable in clinical practice, even with a pre-established DNR order, patients in the DNI group, who all had signed DNR, presented more acceptability to withhold the use of the vasopressor than the patients in EI group (86.1%, 31/36 vs. 34.4%, 11/32, P < 0.001). There was significantly more use of morphine, as a palliative agent to relieve the respiratory distress, in the patients of DNI group, when compare to the EI group (33.3%, 12/36 vs. 12.5%, 4/32, P = 0.04). Between the two groups, there was a significant difference in the length of stay in the ICU (39.97 ± 28.93 vs. 87.66 ± 117.65 hours, P = 0.03); however, there was no significant difference in the total length of hospital stay (58.78 ± 121.02 vs. 92.59 ± 118.32 hours, P = 0.25). The mean cost of hospitalization was also significantly lower for DNI group than for the EI group (New Taiwan Dollars: 61,894.72 ± 38,804.56 vs. 92,775.69 ± 76,668.61, P = 0.046).

**Table 2:** Comparisons between do-not-intubate group and endotracheal intubation group.

Data Field	DNI group (n = 36)	EI group (n = 32)	P value
Male (%)	26 (72.2)	21 (65.6)	0.56
Age (years)	60.44 ± 18.07	54.66 ± 16.40	0.17
Creatinine (mg/dL)	1.96 ± 2.53	2.07 ± 1.89	0.84
AST (IU/L)	55.19 ± 41.98	72.75 ± 87.63	0.29
DNR orders (%)	36 (100)	26 (81.3)	0.007
CPR (%)	0 (0)	6 (18.8)	0.007
MP + CP (%)	33 (91.7)	28 (85.5)	0.57
HP (%)	33 (91.7)	28 (85.5)	0.57
Withhold the use of vasopressor (%)	31 (86.1)	11 (34.4)	<0.001
Morphine use (%)	12 (33.3)	4 (12.5)	0.04
LOS in ICU (hours)	39.97 ± 28.93	87.66 ± 117.65	0.02
LOS in hospital (hours)	58.78 ± 121.02	92.59 ± 118.32	0.25
Costs (NTD)	61894.72 ± 38804.56	92775.69 ± 76668.61	0.04

Abbreviations as Table 1; NTD, New Taiwan dollars

## Discussion

In our study, the patients with respiratory failure had 100% intra-hospital mortality rate, which is similar to the nationwide retrospective cohort study of Taiwan [13]. Once patients poisoned by paraquat progress to respiratory failure, their prognosis is poor, with or without intubation. NBT is defined as an inappropriate life-prolonging treatment, subjecting patients to ongoing and likely uncomfortable conditions, with no direct benefit to the patient [14]. Therefore, intubation of patients with paraquat poisoning can be considered as NBT.

Physicians could prevent patients from undergoing NBT, if parameters were available to predict which patients will develop respiratory failure. Previous studies have shown that plasma and urine paraquat concentration, obtained within the first 24 hours after ingestion, can predict the patient’s outcome [16, 17]. Unfortunately, the plasma paraquat concentration must be measured via spectrophotometry, which is not available at MacKay Memorial Hospital; thus, paraquat poisoning was confirmed by a qualitative urine test. Without a spectrophotometer, a clinical parameter may be used as substitute. The mechanism of paraquat pulmonary toxicity involves the generation of the superoxide anion, which leads to the formation of more toxic reactive oxygen species [18]. Reactive oxygen species are free radicals that can stimulate the release of cytokines and inflammatory mediators from lymphocytes. This results in a systemic inflammatory response, thereby leading to multiple organ dysfunction, including liver and kidney. Yang et al. [6] demonstrated that patients with toxic hepatitis had a greater incidence of respiratory failure than those without toxic hepatitis (63.2% vs. 48.0%, P = 0.037). Kim et al. [19] studied 278 patients with acute paraquat poisoning, from January 2007 to December 2007. The odds ratio for patients with acute kidney injury subsequently developing respiratory failure was 19.6 [95.0% C.I. (8.825, 43.532), P < 0.01], suggesting that an earlier clinical signs of paraquat toxicity, either toxic hepatitis or acute kidney injury, could predict the patient’s progression to respiratory failure. Patients can then be informed about their poor prognosis, and their right to choose DNI, since in this situation intubation is considered an NBT.

A significantly higher number of patients in DNI group used morphine, suggesting that patients in this group understood and accepted their poor prognosis, and these patients expected high-quality palliative and end-of-life care, including the relief of pain and dyspnea. There are no large clinical trials to assess the utility of morphine in patients with paraquat poisoning. However, from the expert opinion and clinical trials of patients with cancer and advanced pulmonary disease, it is reasonable to use morphine as a palliative therapy for patients with paraquat poisoning [20]. It is also possible that patients in EI group used less morphine because their dyspnea and respiratory failure were immediately managed using intubation. With mechanical ventilator support, patients may have been less able to express their uncomfortable dyspnea sensation.

In previous studies, patients who receive palliative care interventions appear to have reduced length of ICU stay [21–24]. Although there was no difference in the length of hospital stay, patients in the DNI group had significantly shorter ICU stay than those in EI group (P = 0.03). It means that patients in the DNI group spent more time in the ward during the whole course of hospitalization. Moreover, patients in EI group spent nearly their entire hospitalization in the ICU. This also explains the lower cost for the DNI group. In addition to the lower daily cost associated with a ward bed, reducing the length of the ICU stay was likely to reduce variable costs such as charges for medicine, laboratory and radiological testing [25]. Furthermore, since the ICU limits the time for visitation, the patients in DNI group may receive more family company at the end of life. In a study of 120 patients in the USA, Greisinger et al. [26] demonstrated that terminally ill patients were concerned about their families, and family support was the essential indicator for the quality of the end of the life.

Overall, intubation in patients with respiratory failure due to paraquat poisoning is an inappropriate life-prolonging treatment. We recommend that physicians should provide patients and their families the choice of palliative care. The palliative care contains the relief of dyspnea, pain, and more humanity and dignity to their end of the life.

## Conclusions

Patients with paraquat poisoning who developed respiratory failure had high hospital mortality. Patients who chose a more palliative therapeutic strategy, which included DNI, spent less time in the ICU and more time with their family, and their hospital medical costs were less.

## Limitations

Our results are limited by factors inherent to single hospital data. Moreover, the retrospective nature of the study and the small patient cohort influenced the certainty of our conclusions. Some laboratory examination, like arteral blood gas analysis, did not be ordered in all of our patients. Further studies are needed to confirm our observations.

## Supporting information

S1 File Paraquat poisoning patients with respiratory failure. (XLSX)
